# Serological Evidence of Subclinical Transmission of the 2009 Pandemic H1N1 Influenza Virus Outside of Mexico

**DOI:** 10.1371/journal.pone.0014555

**Published:** 2011-01-18

**Authors:** Day-Yu Chao, Kuang-Fu Cheng, Tsai-Chung Li, Trong-Neng Wu, Chiu-Ying Chen, Chen-An Tsai, Jin-Hwa Chen, Hsien-Tsai Chiu, Jang-Jih Lu, Mei-Chi Su, Yu-Hsin Liao, Wei-Cheng Chan, Ying-Hen Hsieh

**Affiliations:** 1 Graduate Institute of Microbiology and Public Health, College of Veterinary Medicine, National Chung-Hsing University, Taichung, Taiwan; 2 CMU Biostatistics Center, China Medical University, Taichung, Taiwan; 3 Graduate Institute of Biostatistics, China Medical University, Taichung, Taiwan; 4 Department of Public Health, China Medical University, Taichung, Taiwan; 5 Graduate Institute of Clinical Medical Science, China Medical University, Taichung, Taiwan; 6 Department of Laboratory Medicine, China Medical University Hospital, Taichung, Taiwan; Direccion General de Epidemiologia, Peru

## Abstract

**Background:**

Relying on surveillance of clinical cases limits the ability to understand the full impact and severity of an epidemic, especially when subclinical cases are more likely to be present in the early stages. Little is known of the infection and transmissibility of the 2009 H1N1 pandemic influenza (pH1N1) virus outside of Mexico prior to clinical cases being reported, and of the knowledge pertaining to immunity and incidence of infection during April–June, which is essential for understanding the nature of viral transmissibility as well as for planning surveillance and intervention of future pandemics.

**Methodology/Principal Findings:**

Starting in the fall of 2008, 306 persons from households with schoolchildren in central Taiwan were followed sequentially and serum samples were taken in three sampling periods for haemagglutination inhibition (HI) assay. Age-specific incidence rates were calculated based on seroconversion of antibodies to the pH1N1 virus with an HI titre of 1∶40 or more during two periods: April–June and September–October in 2009. The earliest time period with HI titer greater than 40, as well as a four-fold increase of the neutralization titer, was during April 26–May 3. The incidence rates during the pre-epidemic phase (April–June) and the first wave (July–October) of the pandemic were 14.1% and 29.7%, respectively. The transmissibility of the pH1N1 virus during the early phase of the epidemic, as measured by the effective reproductive number R_0_, was 1.16 (95% confidence interval (CI): 0.98–1.34).

**Conclusions:**

Approximately one in every ten persons was infected with the 2009 pH1N1 virus during the pre-epidemic phase in April–June. The lack of age-pattern in seropositivity is unexpected, perhaps highlighting the importance of children as asymptomatic transmitters of influenza in households. Although without virological confirmation, our data raise the question of whether there was substantial pH1N1 transmission in Taiwan before June, when clinical cases were first detected by the surveillance network.

## Introduction

Since the swine-origin H1N1 influenza virus (S-OIV) was first identified in humans in April, 2009, the virus has caused a widespread illness in many countries worldwide that meets the World Health Organization (WHO) criteria for a pandemic [1], [2]. As this virus contains a unique combination of gene segments from both North American and Eurasian swine lineages, and is antigenically distinct from seasonal human influenza A, a deficiency in protective immunity in persons born after 1957 has been observed, presumably because of their lack of exposure to H1N1 influenza strains that no longer circulated after that time [3], [4]. Prompt action to mitigate the clinical and societal effects of the pandemic was taken by many countries, including surveillance on probable H1N1 cases, airport fever screening, quarantine, and antiviral therapy on probably cases [5]. However, the effectiveness of these interventions remains questionable as the S-OIV human cases were identified in U.S. as early as the last week of March after the first S-OIV case was confirmed in Mexico in March 11 [6], [7]. Relying on surveillance limits the ability to understand the full impact and severity of the epidemic, especially when asymptomatic to mild-symptoms cases are more likely to be present in the early phase before the epidemic occurred [8], [9].

According to US Centers for Disease Control and Prevention (CDC) estimates, more than 1 million people were infected with S-OIV between April 15 and July 24, 2009, leading to 5,011 hospitalizations and 301 deaths in the US [10]. However, reliance on data from routine surveillance to estimate age-specific attack rates during an emerging pandemic is hampered by changes in the sensitivity and specificity of clinical surveillance schemes and the proportion of subclinical infections [10], [11]. Although several serological surveys had been recently conducted, which provided an estimate of the number of people infected with 2009 pandemic H1N1 over time [12], the actual transmission of the virus during different phase of the epidemic can only be estimated by a longitudinal follow-up study.

Although reports of the epidemic in Mexico, the US and Canada has provided important information about the transmissibility of the 2009 pandemic H1N1 virus [7], [13], [14], little is known of the way viruses were transmitted in the community in the early phase before the epidemic. In this report, we provide first serological evidence of early infection by the 2009 pandemic H1N1 viruses outside of Mexico before the imported pandemic H1N1 cases being reported. Compared with surveillance data based on clinical cases and virological investigation, our direct measurement of incidence in different phases (during April–June and July–October) of the epidemic highlights the importance of serology data for providing a novel insight into the epidemiology of 2009 pandemic H1N1 influenza.

## Methods

### Ethics Statement

All subjects in this study gave informed consent and the study was approved by the Medical Ethics Committee of China Medical University with written consent.

### Enrollment of subjects and serological specimens

Since 2007, all schoolchildren in grades 1–4 in Taiwan received a free annual influenza vaccination from the government. In order to evaluate vaccine efficacy, students from elementary schools located in urban (Taichung city) and rural (Nantou county) areas in central Taiwan were recruited for evaluation in a three-year study starting in the fall of 2008, with additional volunteers recruited each successive year. Family members of the students were also recruited to join the study to further the understanding of household transmissions and vaccine effectiveness pertaining to seasonal influenza viruses. The study protocol based on clinical and laboratory data was established including at least two blood samples drawn from the study subjects before and after influenza seasons. Two questionnaire interviews were also conducted by trained interviewers regarding basic demographic and social contact information, in addition to upper respiratory-related symptoms recorded by bi-weekly telephone interview during the influenza season. All subjects gave informed consent and the study was approved by the Medical Ethics Committee of China Medical University.

To evaluate the antibody response against the 2009 pandemic H1N1 virus, only 306 study subjects with three sequential blood samples, taken in the fall of 2008, April–June in 2009 (after the 2008/2009 influenza season), and September–October in 2009 (before the vaccination of both 2009/2010 seasonal and 2009 pandemic influenza strains) were selected. Only information regarding age, sex, geographical area, vaccination status, and collection dates of blood sample were used in this study.

### Laboratory methods

Antibody titres were measured by use of a haemagglutination inhibition (HI) assay following the standard protocol by the WHO [15], [16]. In brief, serum samples were pre-treated with receptor destroying enzyme (RDE, Deka Seriken Co Ltd, Tokyo, Japan) in 1∶4 ratio at 37°C for 16 hours, followed by another 30 minutes at 56°C and after which an equal volume of 1.6% trisodium citrate was added for enzyme inactivation. The different strains of influenza viruses used in this study were first prepared from the culture supernatants of infected Madin-Darby canine kidney (MDCK) cells. 25 microliters (ul) (4 hemagglutination units, HA) of influenza virus was incubated at room temperature for one hour with an equal volume of RDE-treated serum in a V-shape 96-well microtiter plate. After incubation, 25 ul of 1% (vol/vol) chicken red blood cells was added to each well. Hemagglutination inhibition was read after 30 minutes. The virus strain used was originally isolated from the patient infected by S-OIV H1N1, which is antigenically and genetically closely related to A/California/07/2009. To evaluate the cross-reactivity, a vaccine strain of H1N1 (A/Brisbane/59/2007) and the wild-type strain which represented more than 80% of circulating H1N1 during the 2008/2009 influenza season (A/Taiwan/606/2008) were also used. For the HI assay, serum samples were tested with an initial dilution of 1∶10 and a final dilution of 1∶1024, and the titers were expressed as the reciprocal of the highest dilution of serum where hemagglutination was prevented. Samples that were negative by HI were assigned a titre of 1∶5 for the computational purpose of obtaining a geometric mean titre (GMT). We defined seroconversion as a four-fold increase in antibody titers, which was used to calculate the three-month incidence rate later in the statistical analysis.

A set of samples collected during April–June, 2009 with HI titer≥40 was further confirmed by micro-neutralization assay in accordance with WHO standard protocol [15], [16]. In brief, human serum was heat inactivated for 30 minutes at 56°C and two-fold serial dilution were performed in a 50-ul volume of virus growth medium (VGM) starting from 1∶10 in immunoassay plates. The diluted serum was mixed with an equal volume of 100 TCID_50_ per 50 ul of VGM at 37°C for 2 hours. The virus-antibody mixture was added to the 96-well microtiter plate with confluent MDCK cells and incubated for 48 hours at 37°C. At least quadruplicate repeats of each serum samples were performed. Neutralization titer was expressed as the reciprocal serum dilution giving a 50% reduction of the cytotoxic effect.

### Clinical and virological surveillance data sources

Due to the initial novel H1N1 influenza epidemic in Mexico and the increasing number of affected persons in other countries because of traveling, the Taiwanese government set up an influenza pandemic clinical surveillance system that includes airport fever screening followed by laboratory confirmation starting April 28, 2009. The system required that hospitals and clinics report the probable cases determined by the occurrence of at least one of the following conditions: (1) clinically present fever (>38°C), flu-like symptoms, or other flu-related severe diseases such as pneumonia; (2) epidemiologically related, including having direct contact with confirmed or probable cases or having a travel history to countries with confirmed 2009 pandemic H1N1 cases. The system also monitored travelers with fever using ultra-red scan in the airports, and throat swabs were collected from travelers with fever for laboratory confirmation. The government initiated the pandemic H1N1 clinical surveillance system that started April 29 and an increasing number of probable cases was reported, especially after May 15, which correlated with the first imported laboratory-confirmed pH1N1 case on May 19 [17]. The level of the global influenza alert system was raised from phase 5, announced on April 29, to phase 6 on June 11 by the WHO as the number of probable or laboratory-confirmed cases in Taiwan also increased. Due to the fact that all pH1N1-confirmed cases before the end of June were travel-related, we refer to the duration between April-June as the pre-epidemic period. The surveillance system in Taiwan was discontinued after June 18 and replaced by the routine influenza surveillance system, which included the severe cases reporting system and virological surveillance as reported in other publications [18], [19]. The period between July–October was referred to as the epidemic period in our study.

### Statistical analysis

As a haemagglutination inhibition titre above 1∶40 is correlated with protection The three-month incidence rates in two different time periods were calculated based on the new infections among the susceptible population by excluding the persons with antibody titre higher than 1∶40 from the previous blood sampling. The point estimates and 95% point-wise confidence intervals (CIs) of cumulative incidence rates at selected time points between April and June of 2009 were calculated using generalized estimating equation (GEE) with GENMOD procedure in the SAS statistical package version 9.2 (SAS Institute Inc., Cary, NC) to account for correlated data within household. At each selected day, a person is defined as an incidence case if his/her HI has four-fold increase before that day. The reproductive numbers (R_0_) for these two periods with 95% Confidence intervals were also estimated, based on the final size equation of epidemic [20].

## Results

Among the 306 study subjects, 143 were from schoolchildren and their siblings between 5–18 years of age with mean±standard deviation (s.d.) 9.35±2.23 years; 147 were from family adults between 19–60 years of age with mean±s.d. 39.34±6.66 years; and 16 people were older than 60 with mean±s.d. 67.56±4.88 years. The gender distribution was about 1∶1 in the 5–18 age group but about twice and three times as many females as males in the 19–60 and older than 60 age groups, respectively. In all three age groups, there were twice as many study subjects from rural area as those from the urban city. Because of the free vaccination policy for schoolchildren and people over 65 years old, 61.5% and 50% of study subjects voluntarily received 2008/2009 seasonal influenza vaccine in the 5–18 and >60 age groups, respectively. However, only 6.8% of subjects from the 19–60 age groups had been vaccinated (Table 1).

**Table 1:** Cross-reactive antibody response against seasonal influenza vaccine and wild-type strains and pandemic influenza A (H1N1) virus among different age groups.

*Age group*	*5–18*	*19–60*	*>60*
Sample size (N = )	143	147	16
Age(years)			
Mean	9.35±2.23	39.34±6.66	67.56±4.88
Range	5–16	25–60	61–75
Gender			
Male	72(50.3%)	57(38.8%)	4(25%)
Female	71(49.7%)	90(61.2%)	12(75%)
Region			
Urban	49(34.3%)	48(32.7%)	12(75%)
Sub-urban	94(65.7%)	99(67.3%)	4(25%)
Recipients of 08/09 seasonal influenza vaccine	88(61.5%)	10(6.8%)	8(50%)
Increase in antibody titer from the second samples by a factor of ≥4-fold			
Vaccine strain	16(11.2%)	1(0.7%)	0(0%)
Wild-type seasonal strain	13(9.1%)	23(15.6%)	3(18.8%)
Pandemic strain	26(18.2%)	35(23.8%)	5(31.3%)
Vaccine and pandemic strains	4(2.8%)	0(0%)	0(0%)
Seasonal and pandemic strains	5(3.5%)	8(5.4%)	1(6.3%)

In this study, we detected little or no preexisting cross-reactive antibodies against the 2009 H1N1 virus in the 306 samples from all three age groups. Although the GMT of antibodies against the H1N1 vaccine or seasonal strain were significantly high in the sera collected before 2009 among all three age groups, only three had the titre at or above 1∶10, including 2 (12.5%) from the >60 age group and 1 (0.7%) 56 years old grouped with the 19–60 years old (Tables 1 and 2). In the follow-up sera collected from April to June, the overall decrease of the GMT of the antibodies against the H1N1 vaccine or seasonal strain was observed (Table 1). However, 3.5%, 5.4%, and 6.3% of the people from the school-age, adult, and >60 age groups, respectively, were observed having a four-fold increase of HI antibodies against seasonal and 2009 pandemic H1N1 simultaneously. Likewise, 2.8%, 0%, and 0% of these three groups had a four-fold increase of the HI antibodies that simultaneously acts against the vaccine strain and 2009 pandemic H1N1 in the school-aged, adult, and >60 age groups, respectively.

**Table 2:** Proportion of sequential serum samples obtained in different sampling time from the 306-subjects cohort with haemagglutination inhibition titre above the cutoff.

*HI*	*1^st^ sampling time* *		*2^nd^ sampling time*		*Incidence rate (April–June)*		*3^rd^ sampling time*		*Incidence rate (July–October)*	
Age (years)	> = 10	> = 40	> = 10	> = 40†	> = 10	> = 40	> = 10	> = 40	> = 10	> = 40
5–18	0(0%)	0(0%)	33(23.1%)	19(13.3%)	23.1%	13.3%	104(72.7%)	44(30.8%)	66.9%	29.8%
19–60	1(0.7%)	0(0%)	42(28.6%)	21(14.3%)	28.6%	14.3%	121(82.3%)	49(33.3%)	66.7%	29.4%
>60	2(12.5%)	0(0%)	7(43.8%)	3(18.8%)	43.8%	18.8%	16(100%)	4(25%)	76.9%	30.8%
Total	3(0.98%)	0(0%)	82(26.8%)	43(14.1%)	26.8%	14.1%	241(78.8%)	97(31.7%)	71.1%	29.7%

*1^st^ sampling time refer to samples collected in 2008 (after 08–09 seasonal influenza vaccination), 2^nd^ sampling time refer to samples collected during April to June in 2009 (after the 08–09 influenza season) and 3^rd^ sampling time refer to samples collected during September to October in 2009.

† Among those samples with HI titer≥40, 14 (9.8%), 12 (8.2%), and 3 (18.8%) showed neutralization antibody titer>10 in the 5–18, 19–60, and >60 age groups, respectively.

**Figure 1:**
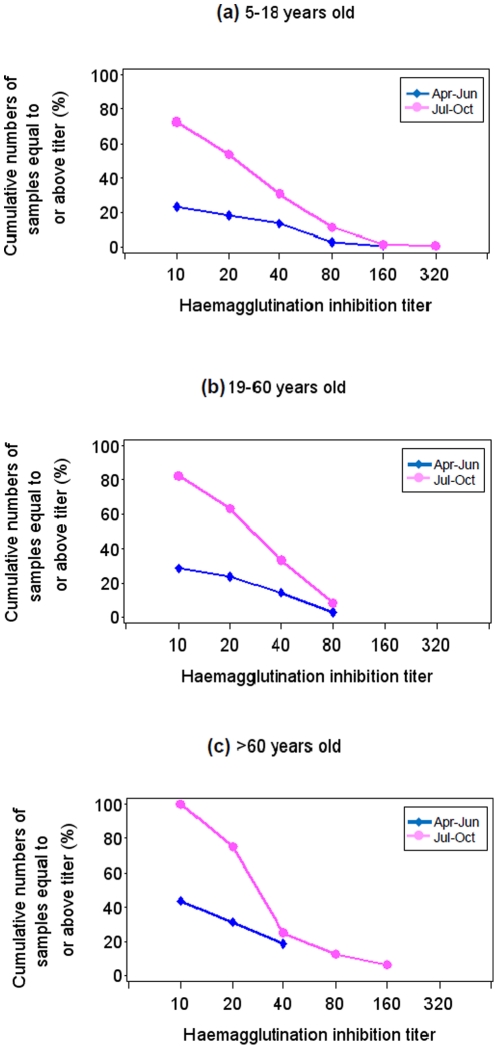
Reverse cumulative distribution curves of antibody titre measured by haemagglutination inhibition assay during April to June and July to October, 2009 among (a) 5–18 age group (b) 19–60 age group (c) >60 age group.

Table 2 shows the proportion of samples in each age group with antibody titre at or above the minimum detection limit (1∶10) or with a titre at least four times the minimum detection limit of the HI assay (1∶40). one of the sera collected during the 1^st^ sampling period presented HI titres greater than 40. Among the sera from the same group collected during the second sampling period (April–June), there were 23.1%, 28.6%, and 43.8% in the respective school-aged, adult, and >60 age groups with HI titres at or above 1∶10. Similar attack rates by 2009 pandemic H1N1 among three age groups (13.3%, 14.3%, and 18.8% from the young, adult, and old age groups, respectively) were observed if HI≥40 was used as the cutoff. Interestingly, significant proportions of blood samples (72.7%, 82.3%, and 100% in the respective young, adult, and older age groups) showed HI titers at or above 1∶10 in the sera collected during the third sampling period (September–October). Again, by using HI≥40 as the cutoff, there was an average of 31.7% having an antibody titer against 2009 pandemic H1N1 among three age groups (30.8%, 33.3%, and 25% from the young, adult and old age groups, respectively). Age-specific reverse cumulative distribution curves of the HI titre from the blood samples collected at different times among the three age groups is given in Figure 1.

**Figure 2:**
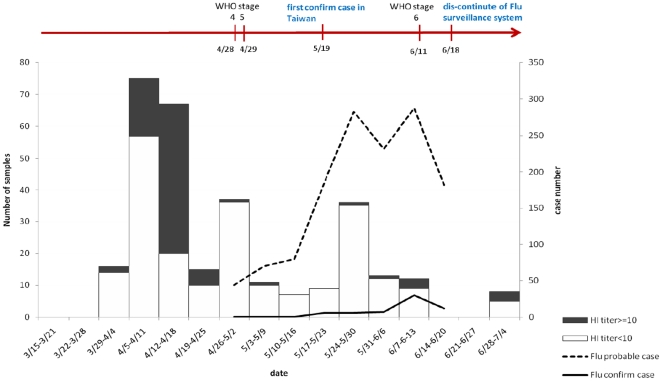
Time course of the early phase before the first wave of 2009 pandemic H1N1. Rectangular bar represented the numbers of sera collected in each week and the antibody titre less than 10 by haemagglutination inhibition assay in white, but in black if equal to or greater than 10. Dash line represented the probably pandemic H1N1 cases fulfilled with the case definition according to the clinical surveillance system. Solid line represented the confirmed H1N1 cases by laboratory confirmation.

The time course of the early phase infection from April to June before the first wave of the 2009 pandemic H1N1 influenza virus is shown in Figure 2.The time distribution of blood samples collected during the first week of April to the last week of June is shown in Figure 2. Surprisingly the earliest time period from nine blood samples with the HI titre against 2009 pandemic H1N1 greater than 40 was between April 5 and 11, which is much earlier than what our clinical H1N1 surveillance system suggested and the first official imported case. Particularly, 4 out of 9 samples were from the children and the rest were from the family contacts.

**Figure 3:**
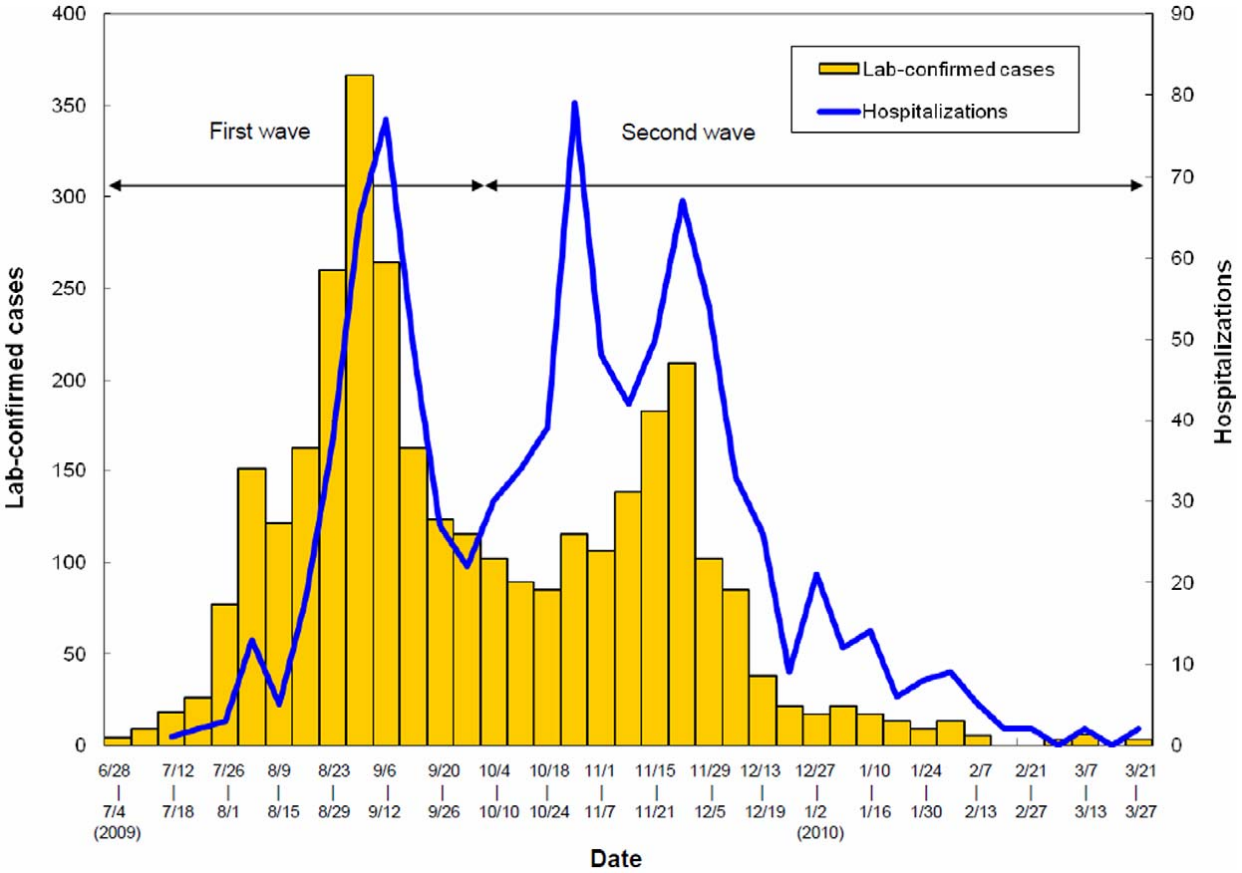
Numbers of hospitalized H1N1 influenza cases and positive H1N1 isolates based on influenza surveillance system in Taiwan. (Source: CDC-Taiwan Novel Influenza A/H1N1 website).

The first wave of 2009 pandemic H1N1 began around July 1 and lasted until the end of September, and was soon followed by the second wave of the fall/winter epidemic with a rapid insurgence of clinically severe influenza cases between October and November that significantly dropped after December, perhaps due to the mass vaccination (Figure 3). The laboratory surveillance data also supported that 90% of the clinical severe cases confirmed to be caused by influenza A were further identified as positive for 2009 pandemic H1N1. It is consistent with our serological finding of intense transmission of the pandemic influenza virus from July to October with an average of 71.1% having an HI titre of greater than or equal to 10 and also an average of 29.7% having an HI titre greater than or equal to 40 (Table 2).

Based on seroconversion (titre turns into 1∶10) or a four-fold increase of the serum titre of the susceptible population in different periods, we conclude that the average incidence rate during the early phase (April–June) of the first wave of the pandemic was 26.8%, ranging from 23.1% in the 5–18 age group to 43.8% in the >60 age group. Meanwhile, the incidence rate during the first wave (July–October) of the pandemic averaged 71.1%. If the HI titre≥40 was determined as seroconversion, the incidence rate during April–June and July–October would be 14.1% and 29.7% on the average, respectively (Table 2).

**Figure 4:**
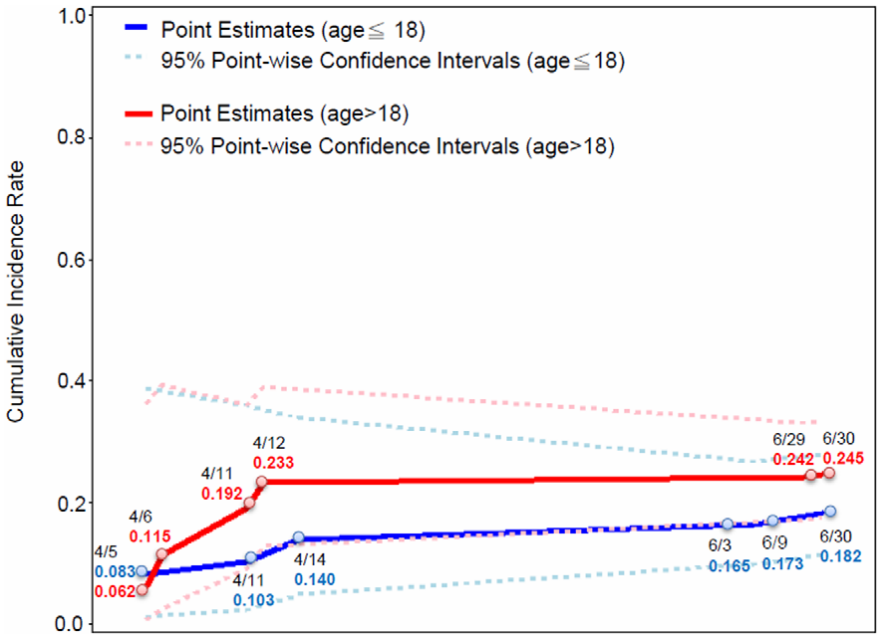
The GEE estimates and 95% point-wise confidence intervals of the cumulative incidence rate during April to June, 2009 for different age groups.

Since the sample size in the >60 age group was too small, the GEE estimate and 95% point-wise CI of the cumulative incidence rate were stratified by the school-age and adult (>18 years old) age groups by combining the adult (19–60) and elderly (>60) age group as shown in Figure 4. The transmission pattern increased smoothly with an incidence rate of 8.3% by April 5, followed by an incidence rate of 14% by April 14, and a gradual increase to 18.2% by the end of June in the school-aged group. However, in the adult age group there was a nearly four-time increase in the cumulative incidence rate from 6.2% by April 5 to 23.3% by April 12 and then remained nearly unchanged with a cumulative incidence rate of 24.5% by the end of June. The transmissibility of 2009 H1N1 pandemic influenza virus during the early phase of the epidemic, as measured by the effective reproductive number R_0_, was 1.16 (95% CI: 0.98–1.34). However, the transmissibility of the virus intensified during the second phase, between July and September, when R_0_ = 1.87 (95% CI: 1.68–2.06).

## Discussion

Our study identified that the earliest blood sampling time period with the HI titer against 2009 pandemic H1N1 greater than 40 was in the three weeks between April 5 and 11, which could be further traced back to the infection period at least before the late of March based on the duration of sero-conversion after infection. Countries in Asia, though far away from the American continent, could be affected as early as the epidemic occurred in Mexico in March as the seroepidemiological studies conducted in Singapore also implied similar findings [21]. Moreover, a recent modeling study in Australia suggests that community transmission of pH1N1 was well established in the state of Victoria in April when the virus was first identified in North America [22]. Therefore, it is plausible the 2009 pandemic influenza virus had spread globally much earlier than any imported cases being detected by the surveillance.

Knowledge of virus-specific immunity in the population and the manner in which it changed as the pandemic progressed is essential for understanding disease transmission. With a direct serological measurement from the sequential specimens of the cohort population, the virus transmission pattern can be estimated from the immunity profile instead of from the clinical attack rate, especially when there may be one-third of asymptomatic or mild infections as reported from the previous volunteer challenge studies [23]. Moreover, most of the studies provide clinical or infection attack rates during the epidemic phase, while our study for the first time in our knowledge gives the immunity profile during the early phase and the first wave of the epidemic. This gave us a better understanding regarding virus transmissibility in different phases of the epidemic as well as its association with clinical surveillance and the effect of control measures including quarantine and surveillance system such as airport fever screening implemented worldwide since May.

Another unique strength of our study is the longitudinal follow-up of the same household cohort which provides the immunity level in different stages of the epidemic. Most studies used the cross-sectional serum samples collected either before or after the epidemic, and the calculation of seroprevalence data would have to be based on a certain cut-off such as an HI titre greater than or equal to 40 without accounting for a pre-immunity status [12], [24], [25]. It is surprising to find a high seroconversion rate during the second and third sampling periods of the epidemic after accounting for the pre-immunity status of the cohort. Our data suggests that the attack rate could be much higher than what we expected if pre-immunity is taken into consideration.

Although the 2009 pandemic H1N1 is antigenically and genetically distinct from haemagglutinins of contemporary human seasonal influenza H1N1 viruses, some degree of cross-reactivity with H1N1 seasonal influenza viruses exists, especially in the older age population as suggested by our data and previous publications [3], [12]. However, the cross-reactive antibody is unlikely to be derived from previous immunization with seasonal influenza vaccine, since such vaccination has been shown to induce little or no cross-reactive antibody to the 2009 pandemic H1N1 virus in any age group [3]. There was a possibility that the sero-positivity of the second blood samples collected during April–June came from cross-reactivity of seasonal H1N1 influenza virus, which circulated in early 2009 in Taiwan as has been documented before. A set of samples collected during April–June, 2009 with HI titer≥40 was further confirmed by the micro-neutralization assay and the result suggested that 9.8%, 8.2%, and 18.8% of the young, adult, and old age groups, respectively, had neutralization titer equal to or greater than 10 against pH1N1, as compared to, respectively, of 13.3%, 14.3%, and 18.8% of the HI titers from the young, adult, and old age groups (Table 2). Although it is still possible that the cross-reactive antibodies present in the serum could still neutralize the virus, our incidence rate during the early phase (April to June) of the epidemic was still quite significant.

Our seropositivity rates by April–June were flat with respect to age (Table 2), while the observation across the world was that for pandemic H1N1 school-age children were most affected with high infection rates whereas few elderly were infected. However, since our cohort was based on a household study design, it may not reflect the infection pattern in the general population. On the other hand, it highlights the important role of children in household transmission of influenza [26], since these adults in our study all live with children and perhaps hence are more likely to be infected at home than adults in general. The lack of virological confirmation also poses a limitation to our study. Also, the gender distribution was about 1∶1 in the 5–18 age group but about twice and three times as many females as males in the 19–60 and older than 60 age groups, respectively (Table 1), perhaps because more females were at home during the blood sampling. Our estimate of the incidence rate during July–October was 29.7% on the average, which was very similar to the previous publications with 21% in the US and 32% in England [12], [24]. Furthermore, the blood samples collected from the populations living in urban and rural areas also suggest a significant difference in seropositivity as suggested previously by Miller et al [12]. Regional variations in seropositivity might be caused by higher transmissions or the higher likelihood of cases being imported to more densely populated regions. Further serum samples during follow-up studies will be tested to document the cumulative incidences of infection by region after the second fall/winter wave of the epidemic, and to investigate whether R_0_ does vary between regions, which might be associated with factors such as population density, household structure, or social contact patterns.

Based on our serological data, the estimate of R_0_ during the early phase before the first wave of the epidemic (Apr–Jun) is 1.16, which increased to 1.87 during the first wave of the epidemic (July–September). The estimate for first wave of the epidemic is very close to the previous publications based on US school outbreaks and Mexico [7], [13], [27]. Whether different generation time, social contacts, or secondary attack rate would apply to the pre-epidemic phase due to virus transmissibility pattern or viral load is a topic for future research [28]–[30].

In summary, our serological results, in combination with the longitudinal study design, show a unique pattern of 2009 pandemic H1N1 infections in different phases of the first wave of the pandemic in Taiwan. This finding should be applicable to other countries that have experienced a similar first wave. Together with the surveillance data, virological surveillance, and serological finding, our study provides valuable insights into the epidemiology of the disease and how this relates to pre-immunity status. This data has been further validated by using Taiwan-CDC pH1N1 hospitalization surveillance data to construct a simple mathematical model to investigate the impact of mass immunization in Taiwan (Hsieh et al. manuscript in preparation). This study, together with the serological data, indicates that the number of infections started to become saturated by mid-November, which suggests that by the time the mass immunization took place in November, the potential for mitigating the overall effect of the second wave by vaccination was relatively limited, perhaps due to population-level immunity acquired from previous infection and intervention such as a class closing policy that had already been in place. Our study will continue to provide the age-specific baseline antibody prevalence and infection rates for specific subtypes (H1, H3 and B) to enhance our understanding of the epidemiology of influenza and its association with the role of transmission played by schoolchildren within households and community, as well as the potential benefits of vaccine protection within household and to the whole population.
