# Correction: Serological Evidence of Subclinical Transmission of the 2009 Pandemic H1N1 Influenza Virus Outside of Mexico

**DOI:** 10.1371/annotation/edeb5a9c-04b2-4d09-ae2b-6d527bf27c81

**Published:** 2011-05-26

**Authors:** Day-Yu Chao, Kuang-Fu Cheng, Tsai-Chung Li, Trong-Neng Wu, Chiu-Ying Chen, Chen-An Tsai, Jin-Hwa Chen, Hsien-Tsai Chiu, Jang-Jih Lu, Mei-Chi Su, Yu-Hsin Liao, Wei-Cheng Chan, Ying-Hen Hsieh

The 7th author's name is misspelled. The correct spelling is: "Jin-Hua Chen"

